# Functional ectopic neuritogenesis by retinal rod bipolar cells is regulated by miR-125b-5p during retinal remodeling in RCS rats

**DOI:** 10.1038/s41598-017-01261-x

**Published:** 2017-04-21

**Authors:** Yan Fu, Baoke Hou, Chuanhuang Weng, Weiping Liu, Jiaman Dai, Congjian Zhao, Zheng Qin Yin

**Affiliations:** 1 Southwest Hospital/Southwest Eye Hospital, Third Military Medical University, Chongqing, 400038 China; 2 Key Lab of Visual Damage and Regeneration & Restoration of Chongqing, Chongqing, 400038 China

## Abstract

Following retinal degeneration, retinal remodeling can cause neuronal microcircuits to undergo structural alterations, which particularly affect the dendrites of bipolar cells. However, the mechanisms and functional consequences of such changes remain unclear. Here, we used Royal College of Surgeon (RCS) rats as a model of retinal degeneration, to study structural changes in rod bipolar cells (RBCs) and the underlying mechanisms of these changes. We found that, with retinal degeneration, RBC dendrites extended into the outer nuclear layer (ONL) of the retina, and the ectopic dendrites formed synapses with the remaining photoreceptors. This ectopic neuritogenesis was associated with brain-derived neurotrophic factor (BDNF) – expression of which was negatively regulated by miR-125b-5p. Overexpression of miR-125b-5p in the retinae of RCS rats diminished RBC ectopic dendrites, and compromised the b-wave of the flash electroretinogram (ERG). In contrast, down-regulation of miR-125b-5p (or exogenous BDNF treatment) increased RBC ectopic dendrites, and improved b-wave. Furthermore, we showed that the regulation of ectopic neuritogenesis by BDNF occurred via the downstream modulation of the TrkB-CREB signaling pathway. Based on these findings, we conclude that ectopic dendrites are likely to be providing functional benefits and that, in RCS rats, miR-125b-5p regulates ectopic neuritogenesis by RBCs through modulation of the BDNF-TrkB-CREB pathway. This suggests that therapies that reduce miR-125b-5p expression could be beneficial in human retinal degenerative disease.

## Introduction

Retinitis pigmentosa (RP) is a form of inherited retinal degeneration that causes blindness in humans, due to a progressive loss of photoreceptors. During retinal degeneration, second-order retinal neurons gradually remodel as a result of the loss of the input signal from photoreceptors, and this remodeling starts with bipolar cells^1^. The Royal College of Surgeon (RCS) rat model is an extensively-studied animal model of this remodeling process. In RCS rats, a mutation in the *MERTK* gene means that retinal pigment epithelial (RPE) cells fail to phagocytose shed photoreceptor outer segments, which leads to progressive photoreceptor degeneration^2^. A previous study from our group has shown that, during retinal degeneration in RCS rats, ectopic dendrites from rod bipolar cells (RBCs) extend into the outer nuclear layer (ONL)^3^. Furthermore, Peng and colleagues have reported that such ectopic RBC dendrites may alter vision immediately, since processing of temporal visual information begins at the dendritic terminals of the bipolar cells^4^. However, the underlying mechanisms of ectopic neuritogenesis remain poorly understood.

MicroRNAs (miRNAs) are a group of short, non-coding RNAs that mediate post-transcriptional gene silencing, and have the potential to be useful targets for the prevention or treatment of retinal degenerative disorders^5^. It has been shown that the expression profile of retinal miRNAs is altered in a mouse model of RP, which suggests that miRNAs are implicated in retinal degeneration^6^. Furthermore, a large pool of different miRNA is expressed in post-mitotic neurons at times of synapse development, and many of these miRNAs are associated with translation regulatory complexes^7, 8^. Two reviews have summarized the properties of thirty miRNAs associated with synapse development and/or plasticity^8, 9^. We hypothesized that miRNAs associated with RP might be involved with the regulation of RBC ectopic neuritogenesis during retinal degeneration. In this study of RCS rats, we used miRNA microarray technology to show that miR-125b-5p was associated with retinal degeneration, and that it regulated dendritic growth and function in RBCs. This work provides proof-of-concept for the potential treatment of retinal degeneration by knockdown of miR-125b-5p.

## Methods

### Animals

The experimental animals were Royal College of Surgeons (RCS) rats and control animals were age-matched RCS-rdy^+^-p^+^ (non-retinal dystrophic) rats. All rats were obtained from the animal center of the Third Military Medical University. All experiments were performed according to protocols approved by the Institutional Review Board of the Third Military Medical University and conformed to the NIH (National Institutes of Health, USA) guidelines on the ethical use of animals.

### Immunostaining

Immunostaining was performed as previously described^10^. Briefly, the enucleated eyecups were immersed in phosphate buffered saline (PBS) containing 4% paraformaldehyde at 4 °C for 2 h and then transferred to 30% sucrose at 4 °C overnight. The eyecups were embedded in optimal cutting temperature (OCT) compound and cut into 10 µm-thick sections. Slices from a distance of 100 µm lateral to the optic nerve were chosen for immunohistochemistry and analysis. The sections were permeabilized with 0.5% Triton X-100 for 15 min and then blocked with 5% goat serum for 1 h. Primary antibodies against protein kinase C alpha (PKCα; 1:200, SC-8393, Santa Cruz Biotechnology, Dallas, TX, USA), C-terminal binding protein 2 (CtBP2; 1:500, sc-5966, Santa Cruz Biotechnology), brain-derived neurotrophic factor (BDNF; 1:1000, ab6201, Abcam, Cambridge, UK), metabotropic glutamate receptor 6 (mGluR6, 1:1500, RA13105, Neuromics, Edina, MN, USA), tropomyosin receptor kinase B (TrkB; 1:200, SC-8316, Santa Cruz Biotechnology), cAMP response element-binding protein (CREB; 1:1000, 9197S, Cell Signaling Technology, Danvers, MA, USA) and cone arrestin (1:500, ab15282, Abcam, Cambridge, UK) were diluted in blocking buffer and incubated at 4 °C overnight. The sections were rinsed three times (15 min each) with PBS at room temperature and incubated at 37 °C for 1 h with secondary antibodies. Nuclei were stained with 4,6-diamidino-2-phenylindole (DAPI; Invitrogen, Carlsbad, CA, US). Fluorescence images were acquired using a Zeiss LSM 700 confocal microscope (Carl Zeiss AG, Oberkochen, Germany).

For quantitative analysis, 5 eyes were sampled in each group and 5 random images of size 213 µm × 213 µm were taken in each retinal slice. We quantified the number of cone cells as the number of cells in the ONL with DAPI-stained nuclei and positive cytoplasmic staining for cone arrestin. The number of rod cells was calculated as the number of cells with DAPI-stained nuclei in the ONL minus the number of cone cells. We quantified the number and length of the ectopic dendrites according to previously described protocols^11^. Dendrites that extended into the ONL were considered to be ectopic dendrites. The fluorescence-labeled ectopic dendrites were manually traced from their initial position on the edge of ONL to their terminus using NeuronJ software^12^. The number and length of ectopic dendrites were then calculated automatically using NeuronJ. To counteract observer (researcher) bias, the image analysis was performed by an individual who was blind to the grouping of the samples.

### Transmission electron microscopy

The retinae were removed from the eyecups and fixed in 0.1 M cacodylate buffer containing 2.5% glutaraldehyde (pH 7.4) for 24 h. The samples were rinsed with PBS several times and then fixed with 1% osmium tetroxide for 2 h. The samples were again rinsed with PBS, dehydrated with increasing concentrations of acetone and embedded in epoxy resin 618. The sections were stained using uranyl acetate and lead citrate. Images were obtained using a TECNAI 10 (FEI-Philips, Hillsboro, OR, USA) transmission electron microscope.

### MicroRNA panel

A microRNA panel assay was performed according to our previous protocol^10^. Briefly, RNA was extracted from the retinae of RCS and control rats using TRIzol (Invitrogen) and the miRNeasy Mini Kit (Qiagen, Hilden, Germany) according to the manufacturers’ instructions. The RNA samples were quantified and labeled using a miRCURY™ Hy3™/Hy5™ Power Labeling kit (Exiqon, Vedbæk, Denmark) and then hybridized using a miRCURY™ LNA Array System (v.18.0). An Axon Instruments GenePix 4000B Microarray Scanner (Molecular Devices, Sunnyvale, CA, USA) was used to acquire digital data of the slides and the data were analyzed using GenePix Pro 6.0 software. A normalization factor was calculated using miRNAs with an intensity ≥30 in three repeated samples, and the expressed data were normalized using median normalization. The relative expressions of thirty synapse-relevant microRNAs were displayed in a heat map.

### Reverse-transcription quantitative polymerase chain reaction (RT-qPCR)

RT-qPCR analysis for miRNAs was performed using a Hairpin-it^TM^ miRNAs qPCR Quantitation Kit (GenePharma, Shanghai, China), according to the manufacturer’s instructions. U6 snRNA was used as an endogenous control for miRNA detection. RT-qPCR analysis for BDNF and mGluR6 mRNA was performed according to previously described methods^13^. Briefly, total RNA was reverse transcribed using a PrimeScript® RT Reagent Kit (Takara Bio USA, Mountain View, CA, USA). Quantitative PCR was performed on a CFX96 Real-Time PCR System (Bio-Rad, Hercules, CA, USA) using a SYBR Green qPCR Mix (Dongsheng Biotech, Guangdong, China) according to the manufacturer’s instructions. The relative expression of BDNF and mGluR6 mRNA was normalized to glyceraldehyde 3-phosphate dehydrogenase (GAPDH). All primers are listed in Table 1.

**Table 1:** Primers for RT-qPCR.

Name	Sequence
miR-125b-5p-F	ACTGATAAATCCCTGAGACCCTAAC
miR-125b-5p-R	TATGGTTTTGACGACTGTGTGAT
U6-F	ATTGGAACGATACAGAGAAGATT
U6-R	GGAACGCTTCACGAATTTG
BDNF-F	GCGCGAATGTGTTAGTGGTTACCT
BDNF-R	AACGGCACAAAACAATCTAGGCTAC
GAPDH-F	GCCCATCACCATCTTCCAGGAG
GAPDH-R	GAAGGGGCGGAGATGATGAC
mGluR6-F	GTGCTAGGTCAACCCTCAAA
mGluR6-R	CTAGAAGAGATCCCAGAGGAGAA
miR-9a-3p-F	GGCGCGGAAATAAAGCTAGATA
miR-9a-3p-R	TATGGTTGTTCACGACTCCTTCAC
miR-124-5p-F	ACTTTCAACGTGTTCACAGCG
miR-124-5p-R	TATGCTTGTTCTCGTCTCTGTGTC
miR-134-5p-F	CCTCTATTCTGTGACTGGTTGACC
miR-134-5p-R	AAAGGTTGATCTCGTGACTCTGTT
miR-219a-5p-F	CTGATTCCCTGATTGTCCAAAC
miR-219a-5p-R	TATGCTTGTTCTCGTCTCTGTGTC
miR-379-5p-F	GCGGCGGGTGGTAGACTATG
miR-379-5p-R	GTGCAGGGTCCGAGGT

### ***In situ*** hybridization and immunostaining

*In situ* RNA hybridization was performed using Basescope technology (Advanced Cell Diagnostics, Hayward, California) following the manufacturer’s protocol with minor modifications. In brief, the frozen retinal sections were treated by Hydrogen Peroxide (Basescope) for 10 min at room temperature, followed by boiling in Target Retrieval (Basescope) for 5 min. After washing with distilled water and 100% ethyl alcohol, Protease Plus (Basescope) was added to the samples for 15 min at room temperature. The sections were rinsed 5 times with distilled water, followed by hybridizing with Basescope probes for 2 h at 40 °C. The slices were then sequentially treated by AMP 0–6 (Basescope). Finally, Fast RED-A and Fast RED-B (Basescope) were mixed to mark the Basescope probes. The slices were then blocked with 5% goat serum for 1 h, and immunostained with primary and secondary antibodies as described above.

### Western blot

For Western blot analysis of whole retinae, retinal samples were lysed in ice-cold radioimmunoprecipitation assay (RIPA) lysis buffer containing protease inhibitors (Beyotime, Nantong, China). The lysates were centrifuged at 15,000 g at 4 °C for 5 min and the supernatants were collected. Protein concentrations were determined using a bicinchoninic acid (BCA) protein assay (Beyotime). A total of 30 μg of protein for each sample was loaded and electrophoresed on a 12% sodium dodecyl sulfate-polyacrylamide gradient gel (SDS-PAGE) and then transferred to polyvinylidene difluoride (PVDF) membranes. The membranes were blocked in 5% non-fat milk at 37 °C for 30 min. The membranes were incubated with anti-BDNF (1:1000, ab6201, Abcam) and anti-GAPDH (1:1000, CW0100, CWBIO) antibodies at 4 °C overnight. The membranes were rinsed with TBST (tris-buffered saline-Tween 20) and incubated with a horseradish peroxidase (HRP)-conjugated secondary antibody (1:1000; Beyotime) at 37 °C for 1 h. The blotted proteins were visualized using an enhanced chemoluminescence (ECL) detection system (Thermo Fisher Scientific, Waltham, MA, USA). Western blot analysis of the retina serial sections was conducted according to previously published methods^14^. Briefly, the retinae were removed from the eyecups, and flattened retinae were gently transferred onto a polyester (PET) membrane with the photoreceptors facing up. To keep the retinae flat, excess liquid was gently removed from underneath the PET membrane. The retinae were placed on a glass slide and frozen at −80 °C for 10 min. The slides were then mounted onto freezing OCT compound and cut into 10 µm serial sections. Total protein was extracted from each section by lysing in ice-cold RIPA lysis buffer (Beyotime) containing protease inhibitors. Western blot was performed as described above, and the primary antibodies used for the retina serial sections included anti-Rhodopsin (1:1000, ab5417, Abcam), anti-TrkB (1:1000, SC-8316, Santa Cruz Biotechnology) and anti-CREB (1:1000, 9197S, Cell Signaling Technology).

### Dual luciferase reporter gene assay

A dual luciferase reporter gene assay was performed according to previously described methods^15^. Briefly, the 3′-UTR region of the rat BDNF mRNA sequence was sub-cloned to the luciferase coding sequence of pmirGLO reporter constructs (Promega, Fitchburg, WI, USA). HEK293T cells were co-transfected with pmirGLO or pmirGLO-BDNF reporter constructs with miR-125b-5p negative control (NC), miR-125b-5p mimics or miR-125b-5p inhibitor using Lipofectamine 2000 (Invitrogen). Each experiment was performed in triplicate. After transfection for 24 h, luciferase activities were measured using a Dual-Luciferase Reporter Assay System (Promega). The data were normalized by dividing the firefly luciferase activity by the Renilla luciferase activity.

### Overexpression of miR-125b-5p

The full-length rno-mir-125b-1 gene was subcloned into the pAAV-CMV-bGlobin-eGFP adeno-associated virus (AAV) vector, which was digested with *Bgl II* and *SalI*. The rno-mir-125b-1 template sequences used were as follows:

UGCGCUCCCCUCAGUCCCUGAGACCCUAACUUGUGAUGUUUACCGUUUAAAUCCACGGGUUAGGCUCUUGGGAGCUGCGAGUCGUGC. The empty AAV vector served as a control (AAV-control). The resultant pAAV-CMV-bGlobin-eGFP-rno-mir-125b-1 AAV vector (AAV-125b) and AAV-control were packaged at Obio Technology Company (Shanghai, China). The packaged AAV was concentrated in PBS at the following titers: AAV-control, 1.96 × 10^13^ and AAV-125b, 2.26 × 10^13^ genome copies per milliliter. Thirteen postnatal day 25 (P25) RCS rats were anesthetized with a mixture of 10 mg/kg ketamine and 1 mg/kg xylazine (intramuscularly; Sigma-Aldrich, St. Louis, MO, USA), and 2 µl of the viral suspension was delivered to the subretinal space using a Hamilton micro-injector.

### Down-regulation of miR-125b-5p

The Tough-Decoy (TuD) miR-125b-5p was subcloned into the pAAV-CAG-eGFP-U6-shRNA vector to generate pAAV-CAG-eGFP-U6-TuD-miR-125b-5p (TuD-125b) for expression of miR-125b-5p antagonists according to a previous protocol^16, 17^. The TuD miR-125b-5p template sequences used were as follows:

GGCGCTAGGATCATCAACTCACAAGTTAGGATCTGTCTCAGGGACAAGTATTCTGGTCACAGAATACAACTCACAAGTTAGGATCTGTCTCAGGGACAAGATGATCCTAGCGCCACCTTTTT. The pAAV-CAG-eGFP-U6-TuDRNA vector served as a control (TuD-control). The packaged viruses were concentrated in PBS at the following titers: TuD-125b, 9.65 × 10^12^ and TuD-Control, 5.06 × 10^12^ genome copies per milliliter. Thirteen P25 RCS rats were anesthetized as above, and 2 μl of the viral suspension was delivered to the subretinal space using a Hamilton micro-injector.

### Subretinal delivery of BDNF

Recombinant human BDNF protein (Catalogue No. 450-02, Peprotech, London, UK) was diluted to 0.2 μg/µl using 0.1 M PBS. Ten P25 RCS rats and 5 P25 control rats were anaesthetized as above, and 2.5 µl of 0.2 µg/µl BDNF or PBS alone (vehicle controls) was delivered to the subretinal space using a Hamilton micro-injector.

### Electroretinogram (ERG)

Dark-adapted animals were prepared for recording under dim red light. The rats were anesthetized as above, and their pupils were dilated with one drop each of tropicamide and phenylephrine. The corneal ERG responses were recorded from both eyes simultaneously using gold wire loops. The reference electrode was inserted under the scleral conjunctiva, and the ground electrode was placed in the tail. Amplification, data acquisition and stimulus presentation were performed using a RETIscan system (Roland Consult, Brandenburg, Germany). The stimulus was delivered at incremental intensities, −4.5, −2.5, −0.5, −0.02, 0.5 and 1 log (cd·s·m^−2^), with a 120 s inter-flash interval.

### Statistics

Data analysis was performed using SPSS 17.0 statistical software (SPSS Inc., Chicago, IL, USA) using an independent two-samples t-test or one-way ANOVA. The data are presented as the mean plus/minus the standard deviation (SD) unless otherwise indicated. p < 0.05 was considered to be statistically significant.

## Results

### Ectopic neuritogenesis of RBCs during retinal degeneration

**Figure 1:**
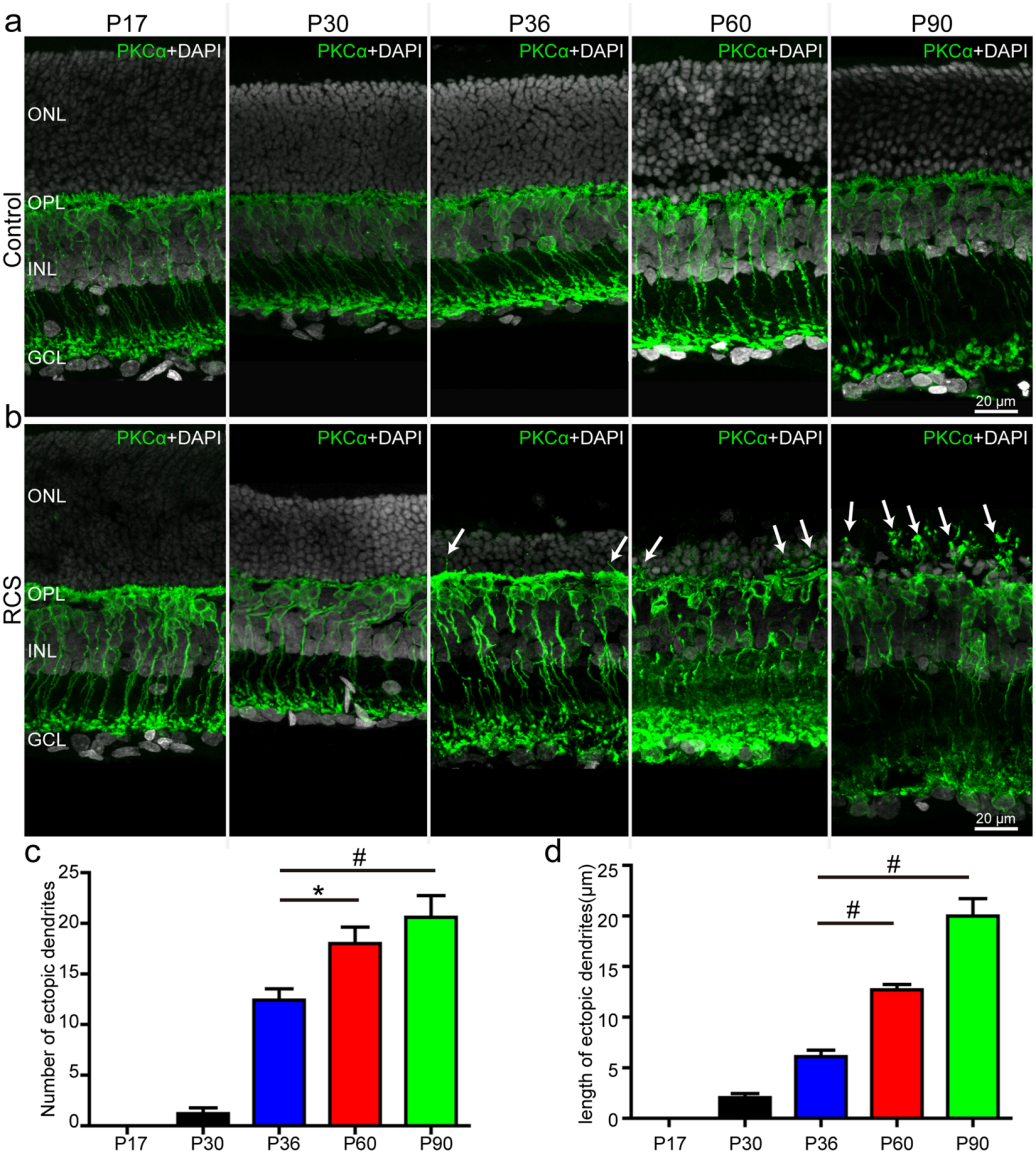
The morphological changes of RBCs during retinal degeneration in RCS rats. Immunostaining for PKCα in the retinae of (**a**) control rats, and (**b**) RCS rats at P17, P30, P36, P60 and P90. The nuclei were stained using DAPI. Arrows indicate RBC dendrites growing into the ONL. (**c**) Number of RBC ectopic dendrites per field view (213 µm × 213 µm) in RCS rat retinae at P17, P30, P36, P60 and P90 (n = 5). (**d**) Same for length of ectopic dendrites (n = 5). ONL, outer nuclear layer; OPL, outer plexiform layer; INL, inner nuclear layer; GCL, ganglion cell layer. Bars show means; error-bars, SEM. *p < 0.05; ^#^p < 0.01.

In the retinae of control rats, the somas of the PKCα-positive RBCs were oval or bottle-shaped, the long axons extended to the inner plexiform layer (IPL), and the dendrites were bushy and candelabrum-like (Fig. 1a). In the retinae of RCS rats, the thickness of the ONL gradually decreased from P17 to P90, which mirrored the progression of retinal degeneration (Fig. 1b). The morphology of the somas and axons of the RBCs in RCS rats appeared normal (the same as controls) from P17 to P90. However, the dendrites of the RBCs in RCS rats were no longer bushy and erect but became flattened (Fig. 1b). Furthermore, some RBC dendrites in RCS rats (which we termed ectopic dendrites^3^) ascended into the ONL from P36 onwards (indicated by the arrows in Fig. 1b). As retinal degeneration advanced, the RBC ectopic dendrites showed increased buckling. In addition, both the number and length of ectopic dendrites gradually increased from P36 to P90 (Fig. 1c and d).

**Figure 2:**
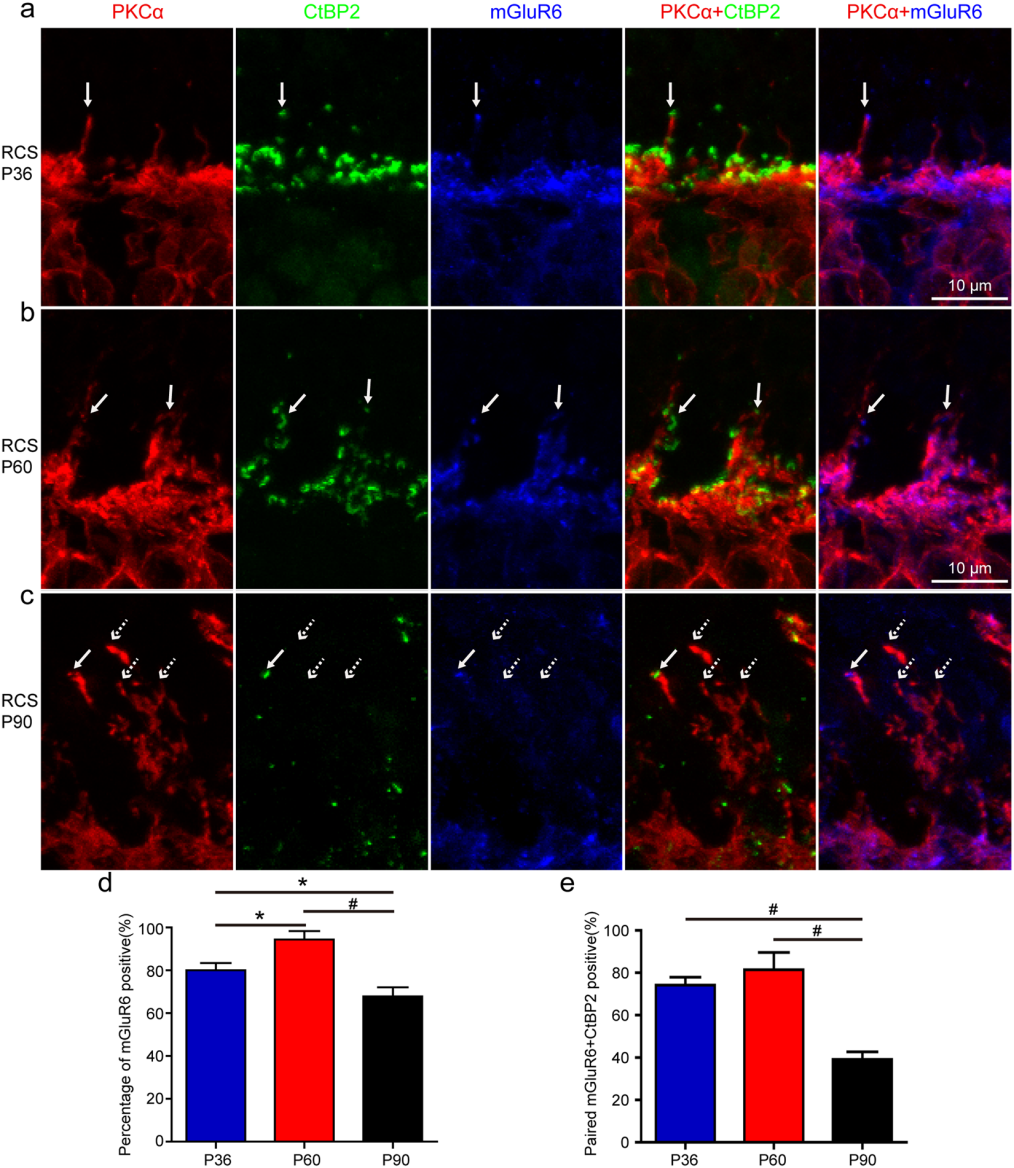
RBC ectopic dendrites in RCS rats formed synapses with the remaining photoreceptors. Immunostaining for PKCα, CtBP2 and mGluR6 in the retinae of (**a**) P36, (**b**) P60 and (**c**) P90 RCS rats. Solid arrows indicate tips of ectopic dendrites that were mGluR6 positive and paired with CtBP2 puncta. Dotted arrows indicate tips of ectopic dendrites that were mGluR6 negative or did not pair with the CtBP2 puncta. (**d**) Proportion of mGluR6-positive RBC ectopic dendrites in RCS rats at P36, P60 and P90, per field view (213 µm × 213 µm) (n = 5) (**e**) Proportion of mGluR6-positive RBC ectopic dendrites paired with CtBP2 in RCS rats per field view (n = 5). Bars show means; error-bars, SEM; *p < 0.05; ^#^p < 0.01.

To determine whether the ectopic dendrites in the ONL retained functional capacity for signal transmission, we performed co-immunolabeling for PKCα, CtBP2 and mGluR6. CtBP2 is a marker of presynaptic ribbons^18^ and mGluR6 is a major functional receptor that is expressed on the ON-RBC dendritic terminals (a postsynaptic marker)^19^. In P36 RCS rats, the tips of 80% ± 3.3% of the ectopic dendrites in the RBCs were mGluR6 positive and 74.2% ± 3.7% of these were adjacent to ectopic CtBP2 positive puncta (indicated by the arrows in Fig. 2a; group quantification in Fig. 2d and e), which suggested that these ectopic dendrites formed synapses with the remaining photoreceptors. Furthermore, electron microscopy showed that the ectopic synaptic structures had typical electron-dense presynaptic ribbons coupled with the postsynaptic compartments in the ONL (Supplementary Fig. S1). In P60 RCS rats, as retinal degeneration advanced, the proportion of mGluR6-positive RBC ectopic dendrites in the RBCs was 94.4% ± 3.9% and the proportion paired with CtBP2 puncta (indicated by the arrows in Fig. 2b) increased significantly to 81.4% ± 8.2%. However, by P90, the proportion of RBCs expressing mGluR6 fell significantly to 67.8% ± 4.2% and the proportion of those juxtaposed to CtBP2 puncta (indicated by the arrows in Fig. 2c) fell to 39.2% ± 3.5%. It is likely that the drop in synaptic contacts between RBC ectopic dendrites and photoreceptors at P60-P90 was simply due to the increasing loss of rods with progressive retinal degeneration. Indeed, in P90 RCS rats, we found that rod cells were completely absent (e.g. Supplementary Fig. S2), and we believed that RBC ectopic dendrites were therefore predominantly forming synapses with the remaining cone photoreceptors. Taken together, these results showed that ectopic dendrites of RBCs formed synapses with the remaining photoreceptors.

### Functional ectopic neuritogenesis was regulated by miRNA during retinal degeneration

**Figure 3:**
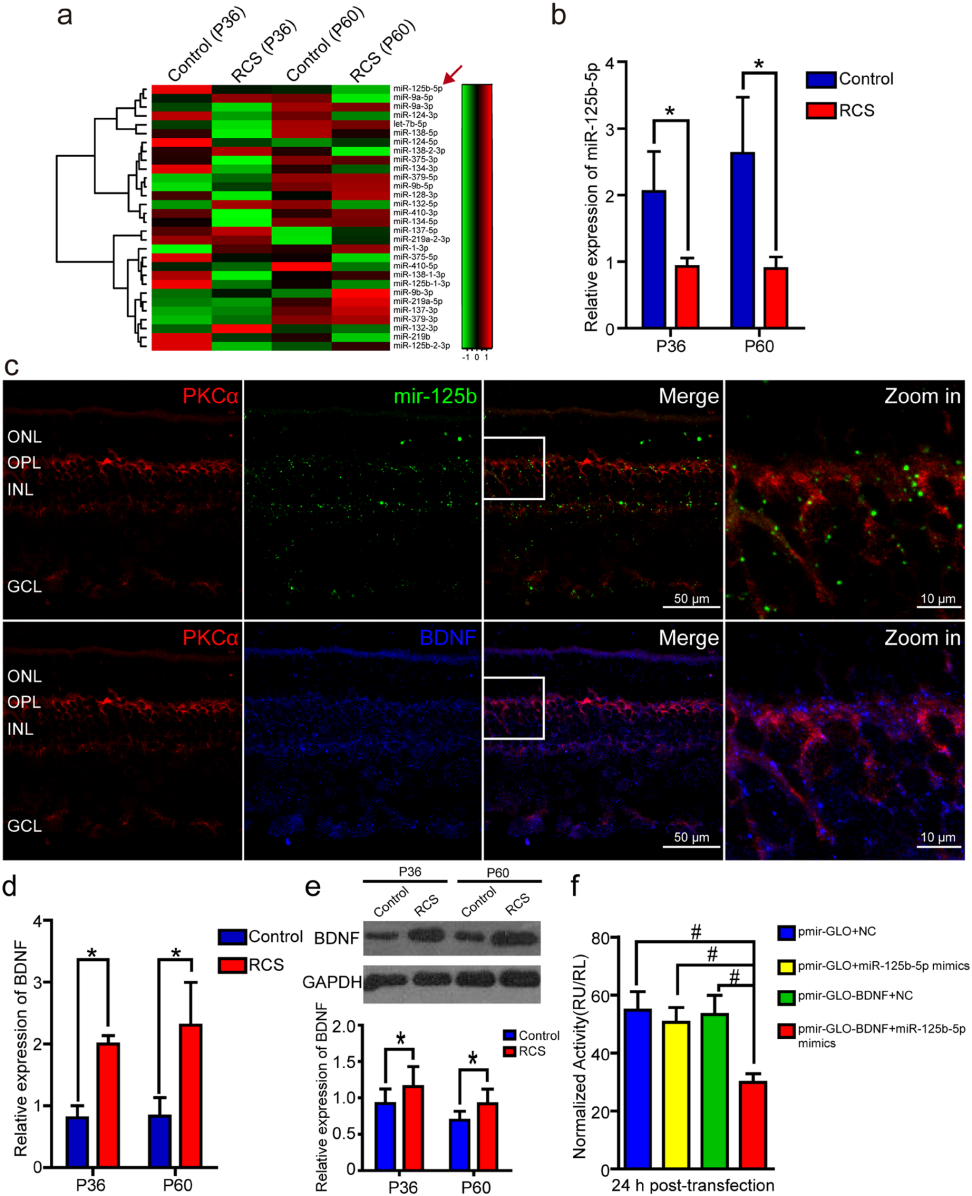
The expression of miR-125b-5p in RBCs and regulation of BDNF mRNA expression. (**a**) Heat map showing the relative expression of 30 synapse-relevant microRNAs in the retinae of the control and RCS rats at P36 and P60. The red arrow indicates where the relative expression of miR-125b-5p in the retinae of RCS rats decreased compared with the control retinae. (**b**) RT-qPCR data showing the relative expression of miR-125b-5p in the retinae of control and RCS rats at P36 and P60 (n = 3). (**c**) Staining for mir-125b, BDNF and PKCα in the retinae of P36 RCS rats. *In situ* hybridization with mir-125b probe (green) was followed by immunostaining with anti-PKCα (red) and anti-BDNF (blue) antibodies, as described in Methods. (**d**) RT-qPCR analysis of the mRNA expression of BDNF in the retinae of control and RCS rats at P36 and P60 (n = 3). (**e**) Western blot analysis of BDNF expression in the retinae of the control and RCS rats at P36 and P60 (n = 3). GAPDH was used as a loading control. For original blot images, please see Supplementary Fig. 11. (**f**) The normalized luciferase activity of pmirGLO (as a control) and pmirGLO-BDNF-transfected cells after negative control (NC) or miR-125b-5p mimic treatment (n = 3). ONL: outer nuclear layer; OPL: outer plexiform layer; INL: inner nuclear layer; GCL: ganglion cell layer. Bars show means; error-bars, SD; *p < 0.05; ^#^p < 0.01.

We performed a microRNA microarray assay to explore the mechanism of ectopic neuritogenesis by RBCs during retinal degeneration in RCS rats. Thirty candidate microRNAs, all previously associated with synaptogenesis^8, 9^, were studied. Of these microRNAs, miR-125b-5p was downregulated most in retinae of RCS rats compared with age-matched control retinae at P36 and P60 (Fig. 3a). We further confirmed that miR-125b-5p was downregulated in the retinae of RCS rats using RT-qPCR (Fig. 3b). However, the other synaptogenesis-associated miRNAs in retinae of RCS rats did not show significant differences from controls at P36 and P60 (Supplementary Fig. S3). To determine the expression of miR-125b-5p in the retinae, we used the recently developed Basescope technology, which allows detection of 50–300 nucleotides (nt) of RNA with very high sensitivity and specificity. Thus, we generated Basescope probes for miR-125b-5p precursor (mir-125b). We found that the mir-125b signal was present in the outer plexiform layer (OPL), inner nuclear layer (INL) and ganglion cell layer (GCL), and was co-localized with PKCα, which suggested that RBCs could be miR-125b-5p positive (Fig. 3c). To our knowledge, this is the first report that combines Basescope assays with immunostaining. Furthermore, BDNF was predicted to be one of targets of miR-125b-5p by TargetScan (Supplementary Fig. S4). Immunostaining showed that BDNF was also expressed in the OPL, INL and GCL, and was co-localized with PKCα (Fig. 3c). RT-qPCR and western blot assays confirmed the expression of BDNF was increased in the retinae of RCS rats (Fig. 3d and e). To validate that miR-125b-5p was directly targeting BDNF, we performed a dual luciferase reporter gene assay, which showed that miR-125b-5p mimics significantly decreased luciferase expression in pmirGLO-BDNF transfected cells (Fig. 3f). In contrast, miR-125b-5p inhibitor significantly increased luciferase expression in the pmirGLO-BDNF transfected cells (Supplementary Fig. S5). We confirmed that neither miR-125b-5p mimics nor inhibitor modulated the luciferase levels in pmirGLO-transfected control cells (Fig. 3f and Supplementary Fig. S5). Taken together, these findings indicated that miR-125b-5p regulated BDNF expression, and suggested that this pathway may be involved in the development of ectopic dendrites in the RBCs of RCS rats.

### Overexpression of miR-125b-5p decreased ectopic dendrites of RBCs

**Figure 4:**
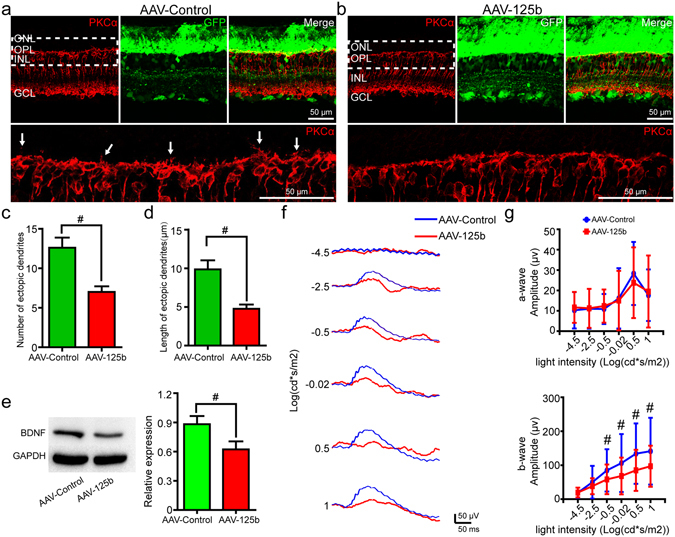
Overexpression of miR-125b-5p decreased the number and length of RBC ectopic dendrites and the ERG responses in RCS rats. Immunostaining of PKCα (red) and GFP (green) in the retinae of P60 RCS rats, five weeks after subretinal injection of (**a**) AAV-control or (**b**) AAV-125b virus. Bottom sub-panel shows magnification of the dotted area in the top left subpanel. Arrows indicate ectopic dendrites. (**c**) Number of RBC ectopic dendrites in RCS rats, five weeks after AAV-Control or AAV-125b treatment, per field view (213 µm × 213 µm) (bars show means; error-bars, SEM; n = 5). (**d**) Same for length of ectopic dendrites (n = 5) (**e**) Western blot analysis of BDNF expression in retinae of RCS rats after AAV-Control or AAV-125b treatment, five weeks post-surgery (bars, means; error-bars, SD, n = 4). GAPDH was used as a loading control. For original blot images, please see Supplementary Fig. 12. (**f**) Representative ERG traces in RCS rats, five weeks after subretinal injection of AAV-control or AAV-125b. (**g**) Group data for the amplitude of a-waves (top) and b-waves (bottom) from ERG recordings in RCS rats, five weeks after surgery (bars, means; error-bars, SD; n = 13). ONL: outer nuclear layer; OPL: outer plexiform layer; INL: inner nuclear layer; GCL: ganglion cell layer. *p < 0.05; ^#^p < 0.01.

To further study the role of miR-125b-5p in the development of ectopic dendrites on RBCs of RCS rats, we next upregulated the expression of miR-125b-5p using AAV-125b. RT-qPCR assays verified that miR-125b-5p was overexpressed in the AAV-125b-injected retinae five weeks after surgery (Supplementary Fig. S6). Immunostaining for PKCα showed a decrease in RBC ectopic dendrites in AAV-125b-injected retinae compared with the control retinae (e.g. Fig. 4a and b). We found that the number of ectopic dendrites in the AAV-125b-injected retinae was significantly decreased compared with controls (Fig. 4c), as was the length of ectopic dendrites (Fig. 4d). RT-qPCR assays also showed that the expression of mGluR6 in AAV-125b-injected retinae was significantly decreased compared with the control retinae (Supplementary Fig. S7). Notably, no other obvious changes were observed, such as any change in the number of photoreceptors at five weeks after overexpression of miR-125b-5p (Supplementary Fig. S8). Western blot showed that overexpression of miR-125b-5p resulted in a decrease in BDNF expression at five weeks after surgery (Fig. 4e). Furthermore, ERG assays were performed to evaluate the functional changes induced by overexpression of miR-125b-5p (e.g. Fig. 4f). These showed that AAV-125b treatment did not affect the a-wave (Fig. 4g), but did significantly decrease the amplitude of the b-wave, five weeks after surgery (Fig. 4g).

### Reduced expression of miR-125b-5p increased ectopic dendrites from RBCs

**Figure 5:**
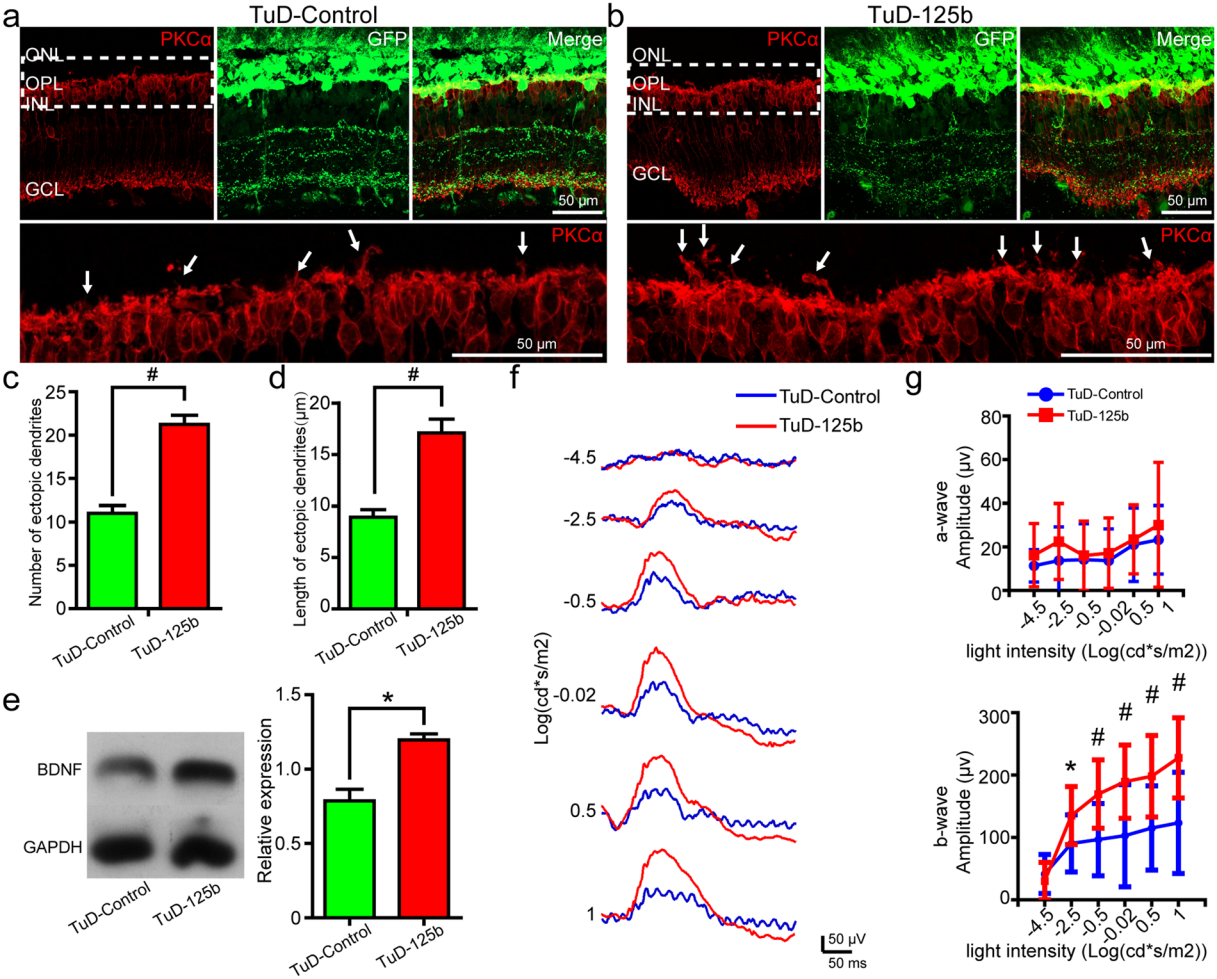
Downregulation of miR-125b-5p increased the number and length of RBC ectopic dendrites and the ERG responses in RCS rats. Immunostaining of PKCα in the retinae of P60 RCS rats, five weeks after subretinal injection of (**a**) TuD-Control or (**b**) TuD-125b virus. Bottom sub-panel shows magnification of the dotted area in the top left subpanel. Arrows indicate ectopic dendrites. (**c**) Number of RBC ectopic dendrites in RCS rats, five weeks after TuD-control or TuD-125b treatment per field view (213 µm × 213 µm) (bars show means; error-bars, SEM; n = 5). (**d**) Same for length of ectopic dendrites (n = 5). (**e**) Western blot analysis of BDNF expression in retinae of RCS rats after TuD-control or TuD-125b treatment, five weeks post-surgery (bars, means; error-bars, SD, n = 4). GAPDH was used as a loading control. For original blot images, please see Supplementary Fig. 13. (**f**) Representative ERG traces in RCS rats, five weeks after subretinal injection of TuD-control or TuD-125b. (**g**) Group data for the amplitude of a-waves (top) and b-waves (bottom) from ERG recordings in RCS rats, five weeks after surgery (bars, means; error-bars, SD, n = 13). ONL: outer nuclear layer; OPL: outer plexiform layer; INL: inner nuclear layer; GCL: ganglion cell layer. *p < 0.05, ^#^p < 0.01.

We next knocked down miR-125b-5p in the retinae of RCS rats using a Tough-Decoy (TuD) approach. The AAV-GFP vectors expressing TuD against miR-125b-5p (TuD-125b) were injected into the subretinal space of P25 RCS rats. RT-qPCR assays validated that miR-125b-5p was down-regulated by TuD-125b five weeks after surgery (Supplementary Fig. S9). In rats injected with TuD-125b, we saw increased ectopic dendrite growth from RBCs, compared to controls (e.g. Fig. 5a,b). Group analysis demonstrated that the number of RBC ectopic dendrites was significantly higher in the TuD-125b group than in TuD-controls (Fig. 5c), as was the length of the ectopic dendrites (Fig. 5d). The expression of mGluR6 was also significantly increased when miR-125b-5p was down-regulated by TuD-125b (Supplementary Fig. S7). Western blot showed that knockdown of miR-125b-5p resulted in an increase in BDNF expression at five weeks after surgery (Fig. 5e). To evaluate the functional changes, ERG assays were performed (e.g. Fig. 5f). These showed that knockdown of miR-125b-5p did not affect the a-wave (Fig. 5g). However, TuD-125b significantly increased the amplitude of the b-wave 5 weeks after surgery (Fig. 5g). Notably, there were also no significant difference in the number of photoreceptors between TuD-125b-injected eyes and control eyes (Supplementary Fig. S8).

### BDNF induced ectopic neuritogenesis by RBCs

**Figure 6:**
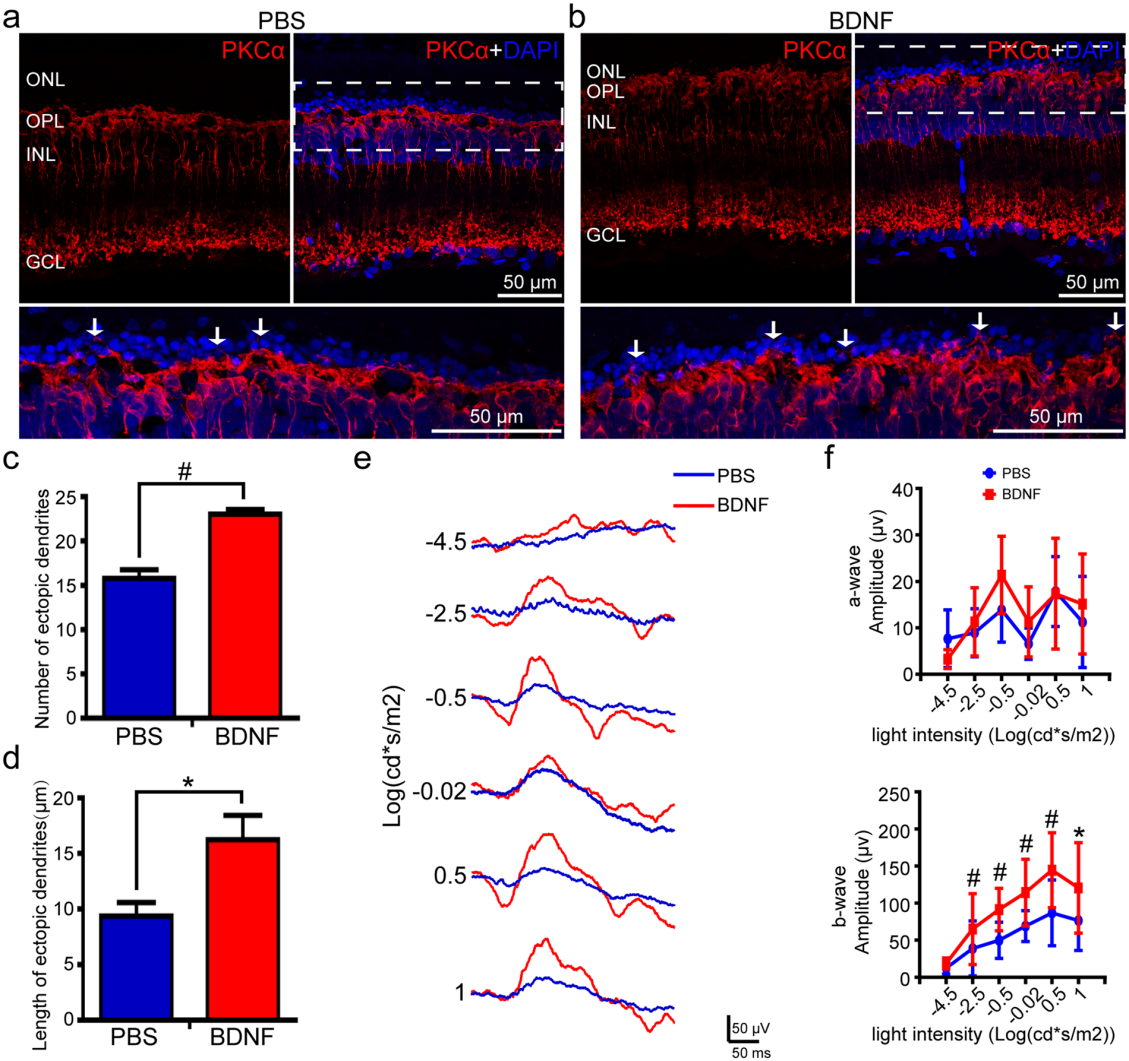
BDNF treatment increased the number and length of RBC ectopic dendrites and the ERG responses in RCS rats. Immunostaining of PKCα in the retinae of P60 RCS rats, five weeks after (**a**) PBS (control) or (**b**) BDNF treatment. Bottom sub-panel shows magnification of the dotted area in the top right subpanel. Arrows indicate ectopic dendrites. (**c**) Number of RBC ectopic dendrites in retinae of RCS rats, five weeks after PBS or BDNF treatment, per field view (213 µm × 213 µm) (bars show means; error-bars, SEM; n = 5). (**d**) Same for length of ectopic dendrites (n = 5) (**e**) Representative ERG traces in RCS rats, five weeks after subretinal injection of PBS or BDNF. (**f**) Group data for the amplitude of a-waves (top) and b-waves (bottom) from ERG recordings in RCS rats, five weeks after surgery (bars, means; error-bars, SD; n = 10). ONL: outer nuclear layer; OPL: outer plexiform layer; INL: inner nuclear layer; GCL: ganglion cell layer. *p < 0.05; ^#^p < 0.01.

The above data showed that reduced expression of miR-125b-5p increased the expression of its target, BDNF, and induced ectopic neuritogenesis by RBCs. To test whether increased levels of BDNF led to ectopic dendrite formation, BDNF was injected subretinally in P25 RCS rats. Five weeks after BDNF treatment, immunostaining for PKCα showed an increase in ectopic dendrites in the RBCs of the BDNF-treated retinae compared with PBS-treated (control) retinae (e.g. Fig. 6a and b). The number of ectopic dendrites in the BDNF-treated retinae was significantly higher than the control retinae (Fig. 6c), as was their length (Fig. 6d). Furthermore, direct BDNF treatment also induced the ectopic neuritogenesis of RBCs in P25 control rats (Supplementary Fig. S10). ERG assays (e.g. Fig. 6e) showed that there were no differences in the amplitudes of the a-wave between the BDNF- and PBS-treated eyes (Fig. 6f). However, the b-wave amplitudes in the BDNF-treated eyes were significantly larger than those in the control eyes at five weeks post-injection (Fig. 6f). In addition, no significant changes were seen in the number of photoreceptors at five weeks after BDNF treatment (Supplementary Fig. S8).

### BDNF induced ectopic neuritogenesis in RBCs via CREB signaling

**Figure 7:**
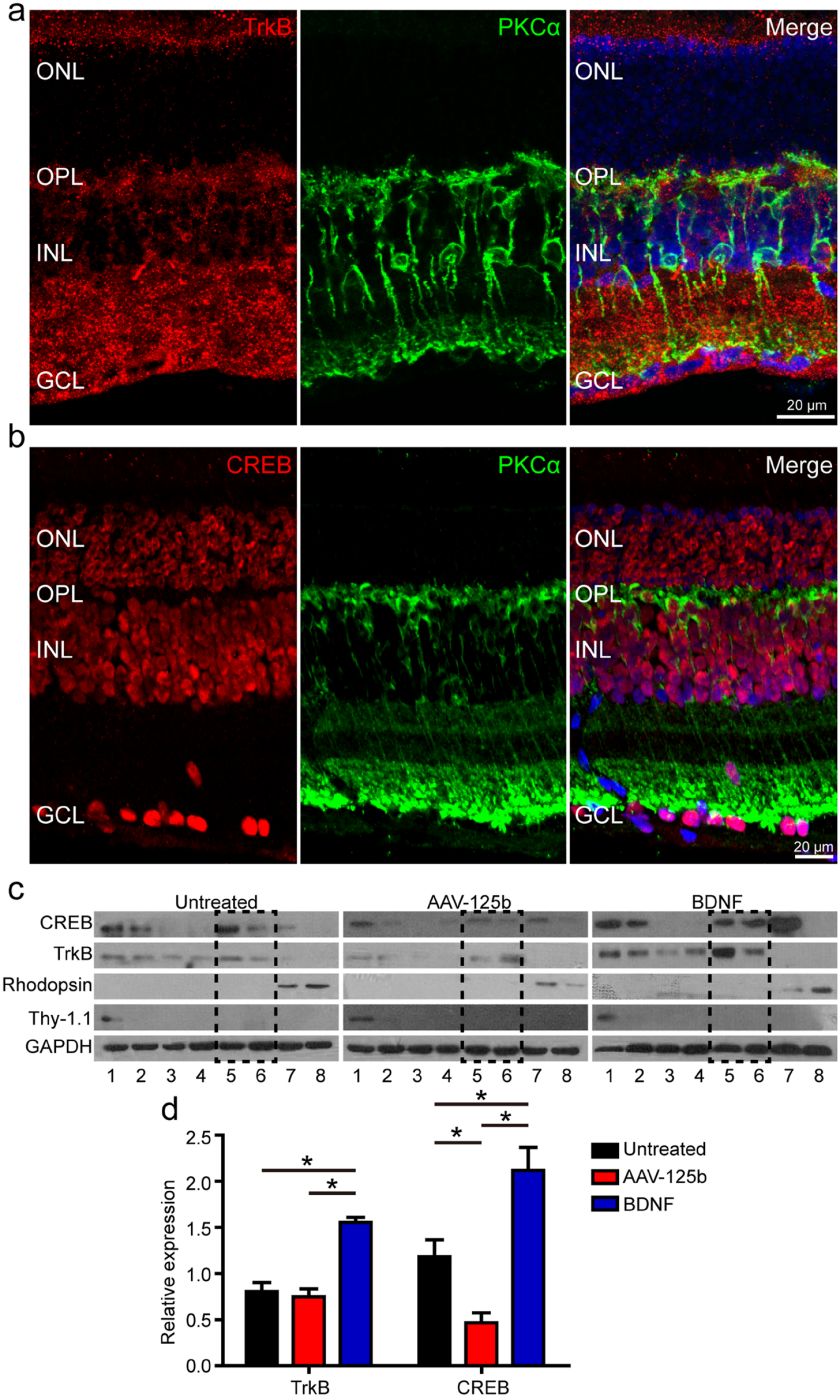
Regulation of RBC ectopic dendrites by the BDNF-TrkB-CREB pathway in RCS rats. (**a**) Immunostaining for TrkB and PKCα in the retinae of P36 RCS rats. (**b**) Immunostaining for CREB and PKCα in the retinae of P36 RCS rats. (**c**) Western blot assay of retinal serial sections in P60 RCS rats, five weeks after AAV-125b or BDNF treatment. The control group consisted of untreated retinae. GAPDH was used as a loading control. The dotted box indicates the position of the OPL and ONL. For original blot images, please see Supplementary Fig. 14. (**d**) The normalized expression of TrkB and CREB in the retinal fractions (bars show means; error-bars, SD; n = 3). ONL: outer nuclear layer; OPL: outer plexiform layer; INL: inner nuclear layer; GCL: ganglion cell layer. *p < 0.05.

From the above findings, we concluded that miR-125b-5p regulated ectopic neuritogenesis in the RBCs of RCS rats via BDNF. Therefore, we next explored the downstream mechanism of BDNF. Firstly, we examined the expression of TrkB (the BDNF receptor) and showed that TrkB and PKCα co-localized, which indicated that the RBCs were TrkB positive (e.g. Fig. 7a). We also examined the expression of CREB, which is a critical transcription factor in the TrkB-activated mitogen-activated protein kinase (MAPK) signaling pathway^20^. As expected, the RBCs were also found to be positive for expression of CREB (Fig. 7b). Western blot was performed to quantify the expression of TrkB and CREB in retinal serial flat sections in RCS rats, five weeks after AAV-125b or BDNF treatment. The retinae were serially cut into a total of eight sections. Thy-1.1 and Rhodopsin antibodies were used to observe the GCL and ONL, respectively. Immunoblot showed that the first section of the flattened retina was positive for Thy-1.1 and the last two sections were positive for Rhodopsin, indicating the locations of the GCL and ONL, respectively (Fig. 7c). Using the immunostaining data for TrkB and CREB, we deduced that the fifth and sixth sections represented the OPL and INL (as indicated by the dotted box in Fig. 7c). As a result, the fifth and sixth sections were used to quantify the expression of TrkB and CREB in the RBCs. Immunoblot showed that overexpression of miR-125b-5p resulted in no change in TrkB expression, but significantly lower expression of CREB (Fig. 7d). In contrast, BDNF treatment induced the increased expression of both TrkB and CREB (Fig. 7d). These findings suggested that BDNF treatment activated the TrkB-CREB pathway, and that this was likely the downstream mechanism by which BDNF regulated ectopic neuritogenesis in RBCs.

## Discussion

Illustration of the early remodeling of retinal circuits is critical for designing therapeutic strategies for the preservation and rescue of vision in patients with retinal degeneration^21^. In the present study, we focused on the function of ectopic dendrites of RBCs, and the molecular mechanisms underlying their development during retinal remodeling. We showed that a specific microRNA, miR-125b-5p, regulated functional dendritic growth in RBCs of RCS rats. Importantly, this work suggests that knockdown of miR-125b-5p could be a therapeutic option for preserving visual function during early retinal degeneration.

During the retinal degeneration of RCS rats, the dendrites of RBC retracted and then sprouted from P17 to P90. This result is in agreement with previous reports^3, 4, 22–24^. Notably, some ectopic dendrites of RBCs were not mGluR6 positive or paired with CtBP2, which suggests that the synaptic connections between ectopic dendrites of RBCs and remnant photoreceptors were formed *de novo*. However, in the *nob2* mouse, aging mouse and in retinal detachment, the outgrowth of RBCs dendrites is triggered by retraction of rod synaptic spherules, in which case pre- and postsynaptic specializations remain juxtaposed, but are displaced, as a unit, to ectopic locations^25–27^. Moreover, the ectopic dendrites of the RBCs in RCS rats formed synaptic connections with cone photoreceptors. Consistent with this, Peng and colleagues have shown, using immune-electron microscopy, that in P35 RCS rats and 10-month-old rhodopsin proline 347 to leucine transgenic (P347L) swine, RBCs dendrites have abnormal (flat-contact type) synaptic contacts with rod and cone terminals^4, 28^. However, there are no ectopic synapses between bipolar cells and photoreceptors if both rods and cones are totally non-functional, which suggests that these connections represent an effort by bipolar cells to restore their input activity^21, 29^. Indeed, the synaptic function of the RBCs may persist despite re-wiring^30^. Accordingly, we suggest that the ectopic dendrites of RBCs seen in this study may form to preserve some functional capacity for signal transmission in the degenerative retina. Using gain- and loss-of-function experiments, we showed that miR-125b-5p was involved in the regulation of ectopic dendrite formation by the RBCs of RCS rats, and that this regulation occurred via BDNF signaling. Overexpression of miR-125b-5p in RCS rats inhibited the expression of BDNF and the RBC ectopic dendrites were decreased. In contrast, knockdown of miR-125b-5p (or subretinal injection of BDNF) induced RBC ectopic dendrite formation. Although miR-125b-5p is highly expressed in the retina and is involved in retinal development^31, 32^, this is the first study to demonstrate that miR-125b-5p regulates the development of ectopic dendrites on RBCs during retinal degeneration. Recently, miR-125b has been shown to modulate synaptic structure and function through the regulation of the NMDA receptor subunit, NR2A^33^. Given that RBCs lack NMDA receptors, this is unlikely to be the mechanism of neuritogenesis by RBCs of RCS rats. This suggests that miR-125b regulates synaptic structure and function in different kinds of neurons via different downstream effectors.

It is commonly accepted that the a-wave of ERG originates from the light response of photoreceptors and that the b-wave represents the activity of the bipolar cells^34, 35^. Since miR-125b-5p is mainly expressed in the OPL, INL and GCL, photoreceptors are not regulated by miR-125b-5p. Consistent with this, neither manipulation of miR-125b-5p, nor BDNF treatment, affected the number of photoreceptors and the ERG a-wave. However, overexpression of miR-125b-5p in RCS rats decreased b-wave amplitude, and conversely, knockdown of miR-125b-5p (or subretinal injection of BDNF) improved b-wave amplitude. This suggests that the observed changes in the ectopic dendrites of RBCs are accompanied by changes in the activity of RBCs. Perhaps more importantly, this also suggests that knockdown of miR-125b-5p, or subretinal injection of BDNF, could potentially rescue vision in retinal degeneration.

Kwon and colleagues have demonstrated that BDNF shapes the dendrites of hippocampal neurons through its receptor, TrkB, and the downstream regulator, CREB^36^. Previous retinal studies have shown that RBCs are TrkB positive^37–39^, suggesting that the BDNF-TrkB pathway regulates RBCs. In this study, we showed that RBCs in RCS rats were both TrkB and CREB positive (although TrkB and CREB are not specific for RBCs). Moreover, BDNF treatment activated the expression of TrkB and CREB and the number and length of RBC ectopic dendrites increased, which suggests that BDNF-TrkB-CREB pathway was involved in the ectopic neuritogenesis. However, the BDNF-TrkB-CREB pathway may not be the critical factor for regulating ectopic dendrite development in RBCs. Studies have shown that knockout of BDNF or TrkB does not affect the development of dendrites in RBCs (although TrkB knockout completely inhibits signaling between rod cells and the RBCs)^37, 40^. This apparent discrepancy is most likely due to different regulatory mechanisms between the development phase and the post-development phase of the retina.

In conclusion, we have shown in RCS rats, functional ectopic neuritogenesis by RBCs is regulated by miR-125b-5p during retinal remodeling. Knockdown of miR-125b-5p increases ectopic dendrite formation by RBCs, and can improve the ERG b-wave, via increased BDNF expression. The results from this study not only improve understanding of the molecular mechanisms of retinal remodeling, but suggest a new treatment for retinal degeneration: miR-125b-5p knockdown. However, miR-125b-5p and BDNF are not uniquely expressed in RBCs, and further studies are therefore necessary to determine if miR-125-5p and BDNF are involved in other retinal remodeling processes.

## Electronic supplementary material

Functional ectopic neuritogenesis by retinal rod bipolar cells is regulated by miR-125b-5p during retinal remodeling in RCS rats
