# Supplementary material for: Functional ectopic neuritogenesis by retinal rod bipolar cells is regulated by miR-125b-5p during retinal remodeling in RCS rats
Source: Sci Rep. 2017 Apr 21;7:1011. doi: 10.1038/s41598-017-01261-x (PMC5430652; doi:10.1038/s41598-017-01261-x)
Supplement: Supplementary file 1 — Functional ectopic neuritogenesis by retinal rod bipolar cells is regulated by miR-125b-5p during retinal remodeling in RCS rats [file 41598_2017_1261_MOESM1_ESM.pdf]

Functional ectopic neuritogenesis by retinal rod bipolar cells is regulated by miR-125b-5p during retinal remodeling in RCS rats

Yan Fu, Baoke Hou, Chuanhuang Weng, Weiping Liu, Jiaman Dai, Congjian Zhao*, Zheng Qin Yin*

SUPPLEMENTARY INFORMATION

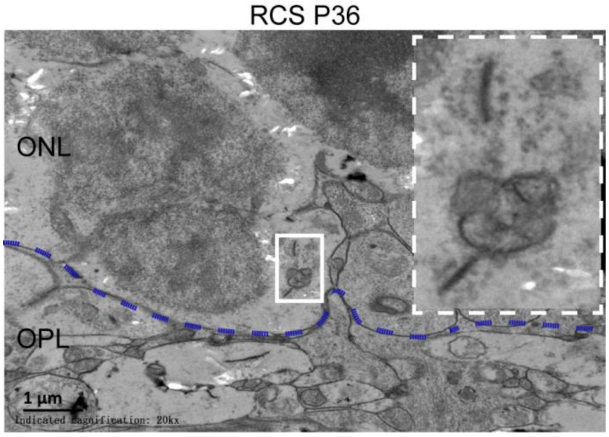

Supplementary Figure S1. Representative transmission electron micrograph of retina in a P36 RCS rat. Blue dotted line shows the edge of ONL. Inset shows high magnification of box in main panel. ONL: outer nuclear layer; OPL: outer plexiform layer.

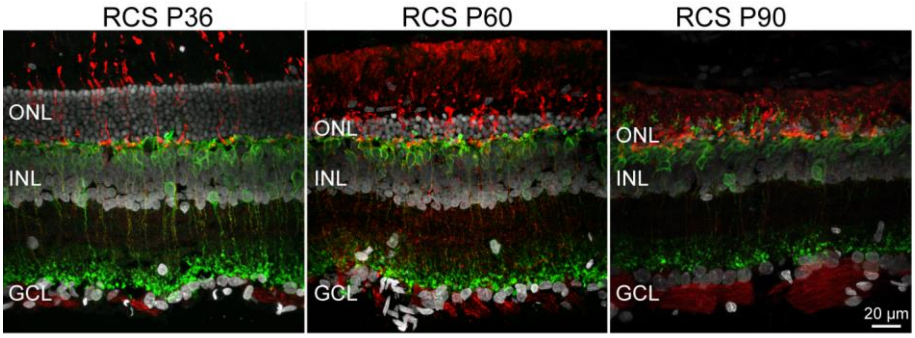

Supplementary Figure S2. Representative images showing immunostaining for

PKCα (green) and cone arrestin (a marker for cone photoreceptors) (red) in the retinae of RCS rats at P36, P60 and P90. Nuclei were stained using DAPI. ONL: outer nuclear layer; INL: inner nuclear layer; GCL: ganglion cell layer.

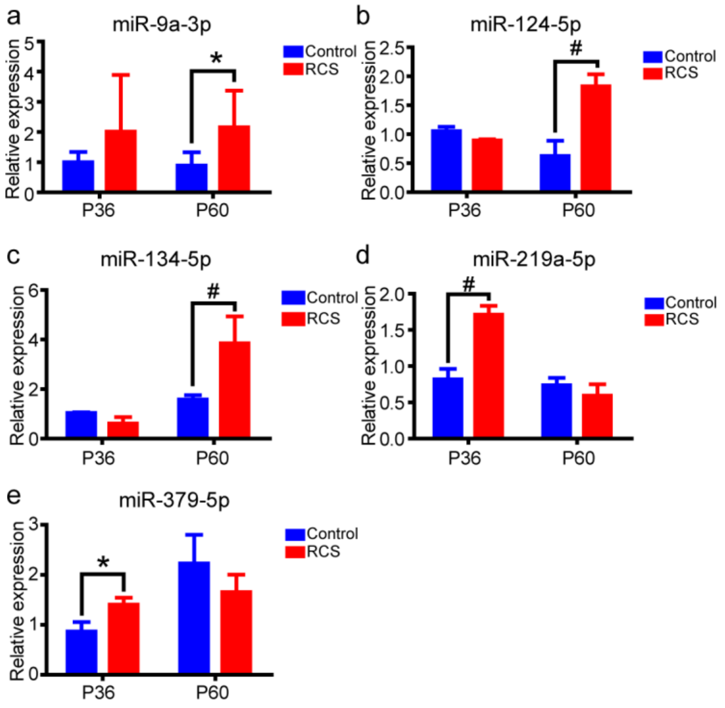

Supplementary Figure S3. RT-qPCR validation of the expression of synapse-relevant miRNAs in the retinae of control and RCS rats. (bars show means; error-bars, SD; n=3). #, p<0.01; *, p<0.05.

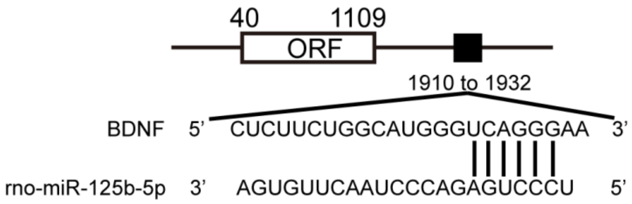

Supplementary Figure S4. Putative binding sites of miR-125b-5p in the 3'-UTR of BDNF mRNA.

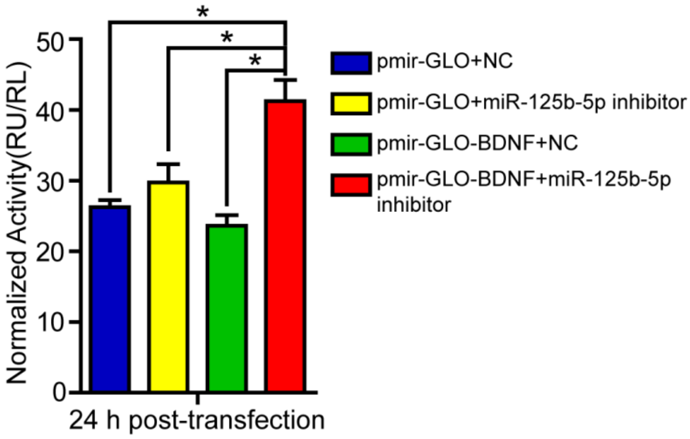

Supplementary Figure S5. The normalized luciferase activity of pmirGLO (as a control) and pmirGLO-BDNF transfected cells after negative control (NC) or miR-125b-5p inhibitor treatment (bars show means; error-bars, SD, n=3). *, p<0.05.

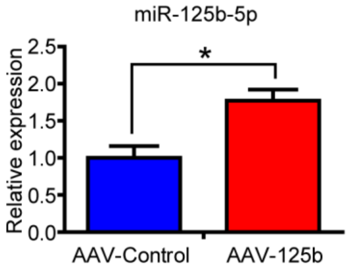

Supplementary Figure S6. Relative expression of miR-125b-5p in AAV-control and AAV-125b treated retinae (bars show means; error-bars, SD, n=3). *, p<0.05.

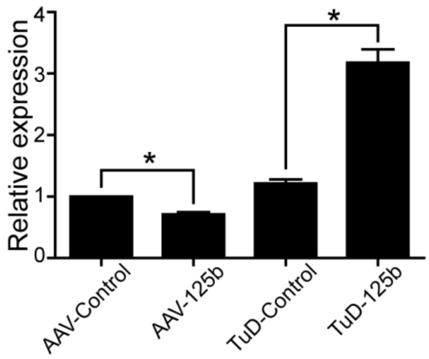

Supplementary Figure S7. Relative expression of mGluR6 in the AAV-control, AAV-125b, TuD-control and TuD-125b treated retinae (bars show means; error-bars,

SD, n=3). *, p<0.05.

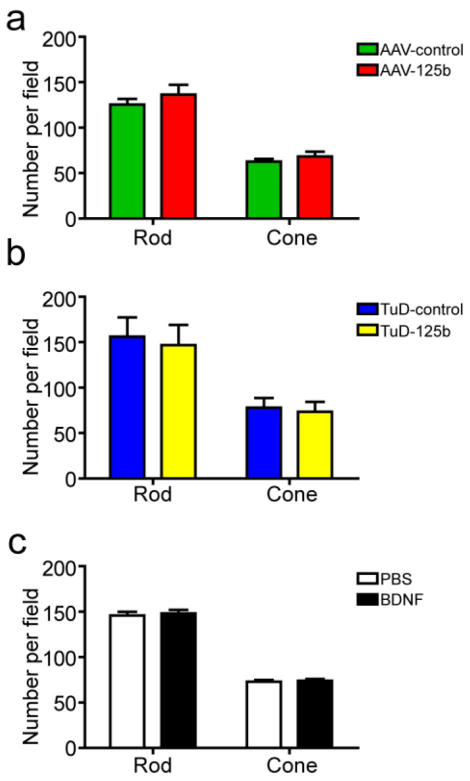

Supplementary Figure S8. Number of rod and cone in retinae of RCS rats, five weeks after AAV-125b, TuD-125b and BDNF treatment per field view (213 μm × 213 μm) (bars show means; error-bars, SEM; n=5). AAV-control, TuD-control and PBS served as controls.

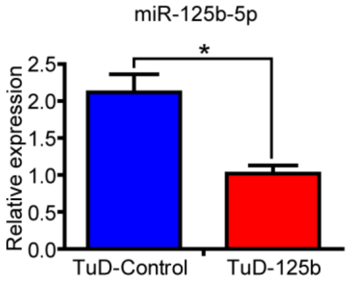

Supplementary Figure S9. Relative expression of miR-125b-5p in the TuD-control and TuD-125b treated retinae (bars show means; error-bars, SD, n=3). *, p<0.05.

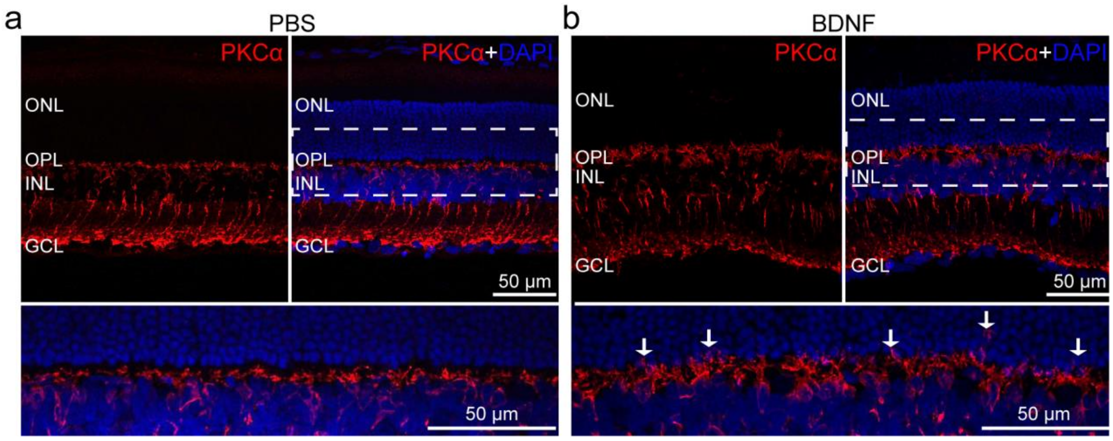

Supplementary Figure S10. BDNF treatment increased the number of RBC ectopic dendrites in control rats. Immunostaining of PKCα in the retinae of P60 control rats, five weeks after (a) PBS or (b) BDNF treatment. Bottom sub-panel shows magnification of the dotted area in the top right subpanel. Arrows indicate ectopic dendrites. ONL: outer nuclear layer; OPL: outer plexiform layer; INL: inner nuclear layer; GCL: ganglion cell layer.

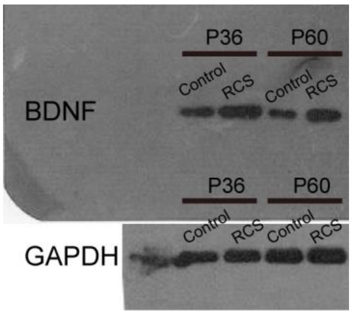

Supplementary Figure S11. Original file of Western blot analysis of BDNF expression in the retinae of the control and RCS rats at P36 and P60.

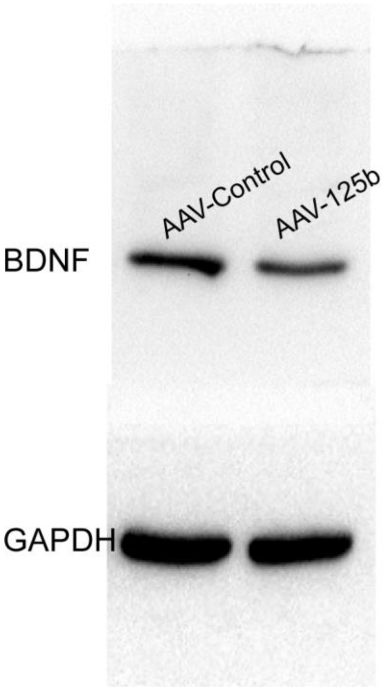

Supplementary Figure S12. Original file of Western blot analysis of BDNF expression in retinae of RCS rats after AAV-Control or AAV-125b treatment, five weeks post-surgery.

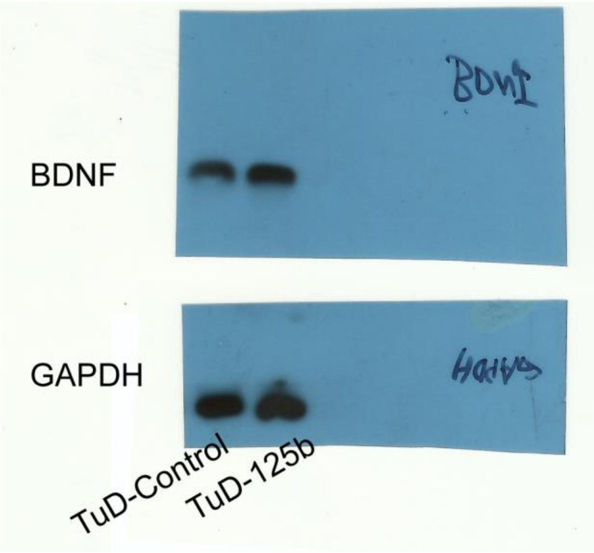

Supplementary Figure S13. Original file of Western blot analysis of BDNF expression in retinae of RCS rats after TuD-control or TuD-125b treatment, five weeks post-surgery.

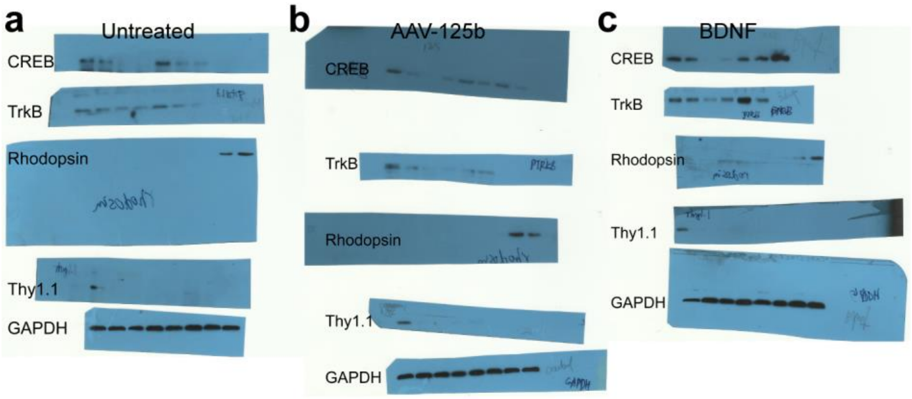

Supplementary Figure S14. Original file of Western blot assay of retinal serial sections in P60 RCS rats, five weeks after AAV-125b or BDNF treatment.
